# *N*-(5-Amino-2-methyl­phen­yl)-4-(3-pyri­dyl)­pyrimidin-2-amine

**DOI:** 10.1107/S1600536810000899

**Published:** 2010-01-13

**Authors:** Jerry P. Jasinski, Ray J. Butcher, Q. N. M. Hakim Al-Arique, H. S. Yathirajan, B. Narayana

**Affiliations:** a Department of Chemistry, Keene State College, 229 Main Street, Keene, NH 03435-2001, USA; b Department of Chemistry, Howard University, 525 College Street NW, Washington, DC 20059, USA; c Department of Studies in Chemistry, University of Mysore, Manasagangotri, Mysore 570 006, India; d Department of Studies in Chemistry, Mangalore University, Mangalagangotri 574 199, India

## Abstract

The title compound, C_16_H_15_N_5_, crystallizes with two independent mol­ecules (*A* and *B*) in the asymmetric unit. The dihedral angles of the pyrimidine ring with the benzene and pyridyl rings are 22.3 (1) and 53.2 (9)°, respectively, in mol­ecule *A*, and 6.8 (1) and 11.6 (9)° in mol­ecule *B*. The crystal packing is influenced by the collective action of weak inter­molecular N—H⋯N hydrogen bonds, a π–π stacking inter­action between neighbouring pyridyl rings of mol­ecule *A* [centroid–centroid distance = 3.8395 (10) Å] and C—H⋯π inter­actions.

## Related literature

For imatinib mesylate, see: Druker *et al.* (1996 ▶, 2001 ▶); Kalaycio (2004 ▶); Peggs & Mackinnon (2003 ▶). For related structures, see: Hu *et al.* (2006 ▶); Lynch & McClenaghan (2001 ▶); Santoni *et al.* (2008 ▶); Wolska *et al.* (2003 ▶). For bond-length data, see: Allen *et al.* (1987 ▶). For MOPAC Parameterized Model 3 calculation, see: Schmidt & Polik (2007 ▶).
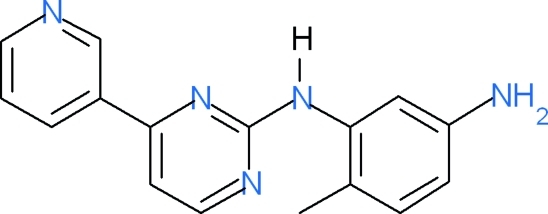


## Experimental

### 

#### Crystal data

C_16_H_15_N_5_
*M* *_r_* = 277.33
Triclinic, 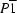

*a* = 9.2242 (3) Å
*b* = 12.5399 (4) Å
*c* = 12.8594 (4) Å
α = 72.719 (3)°
β = 89.724 (3)°
γ = 77.712 (3)°
*V* = 1385.05 (8) Å^3^
*Z* = 4
Cu *K*α radiation
μ = 0.67 mm^−1^
*T* = 200 K
0.55 × 0.48 × 0.37 mm

#### Data collection

Oxford Diffraction Gemini R diffractometer
11476 measured reflections
5337 independent reflections
4629 reflections with *I* > 2σ(*I*)
*R*_int_ = 0.041

#### Refinement

*R*[*F* ^2^ > 2σ(*F* ^2^)] = 0.066
*wR*(*F* ^2^) = 0.198
*S* = 1.08
5337 reflections
381 parameters
H-atom parameters constrained
Δρ_max_ = 0.32 e Å^−3^
Δρ_min_ = −0.36 e Å^−3^

### 

Data collection: *CrysAlis PRO* (Oxford Diffraction, 2007 ▶); cell refinement: *CrysAlis PRO*; data reduction: *CrysAlis PRO*; program(s) used to solve structure: *SHELXS97* (Sheldrick, 2008 ▶); program(s) used to refine structure: *SHELXL97* (Sheldrick, 2008 ▶); molecular graphics: *SHELXTL* (Sheldrick, 2008 ▶); software used to prepare material for publication: *SHELXTL*.

## Supplementary Material

Crystal structure: contains datablocks global, I. DOI: 10.1107/S1600536810000899/is2512sup1.cif

Structure factors: contains datablocks I. DOI: 10.1107/S1600536810000899/is2512Isup2.hkl

Additional supplementary materials:  crystallographic information; 3D view; checkCIF report

## Figures and Tables

**Table 1:** Hydrogen-bond geometry (Å, °)

*D*—H⋯*A*	*D*—H	H⋯*A*	*D*⋯*A*	*D*—H⋯*A*
N1*A*—H1*AA*⋯N5*B*^i^	0.88	2.36	3.154 (2)	151
N2*A*—H2*AB*⋯N3*A*^ii^	0.88	2.11	2.9815 (17)	170
N1*B*—H1*BA*⋯N5*A*^i^	0.88	2.31	3.130 (2)	155
C2*A*—H2*AA*⋯*Cg*3	0.95	2.87	3.7834 (19)	162
C14*A*—H14*A*⋯*Cg*2^iii^	0.95	2.64	3.4709 (18)	146

Symmetry codes: (i) 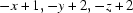; (ii) 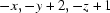; (iii) 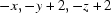. *Cg*2 and *Cg*3 are centroids of the C1*A*–C6*A* and C1*B*–C6*B* rings, respectively.

**Fig. 1.**
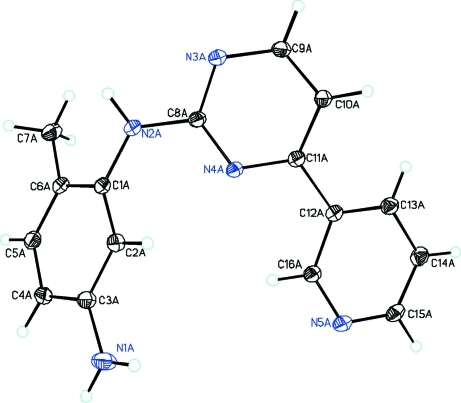
Molecular structure of (I), C16H15N5, molecule A, showing the atom labeling scheme and 50% probability displacement ellipsoids.

**Fig. 2.**
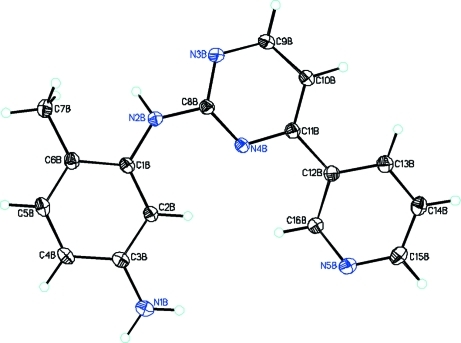
Molecular structure of (I), C16H15N5, molecule B, showing the atom labeling scheme and 50% probability displacement ellipsoids.

**Fig. 3.**
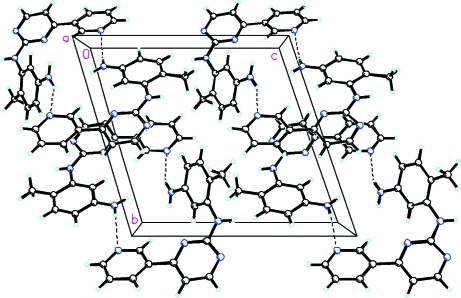
The molecular packing for (I) viewed down the a axis. Dashed lines indicate weak N—H···N intermolecular hydrogen bond interactions which link the molecule into chains propagating along the [011].

## supplementary crystallographic information

### Comment

The title compound, (I), C_16_H_15_N_5_, is an intermediate in the synthesis of imatinib mesylate, a specific inhibitor of Bcr-Abl kinase, produces clinical remission in CML patients with minimal toxicity (Druker *et al.*, 1996, 2001). This selective inhibition of Bcr-Abl kinase by STI-571 has been a successful therapeutic strategy for CML because of the high efficacy and mild side effects of this compound (Peggs & Mackinnon, 2003; Kalaycio, 2004). Imatinib, a 2-phenylaminopyrimidine derivative that functions as a specific inhibitor of a number of tyrosine kinase enzymes, is a drug used to treat certain types of cancer and is used in treating chronic myelogenous leukemia (CML), gastrointestinal stromal tumors (GISTs) and a number of other malignancies. It is the first member of a new class of agents that act by inhibiting particular tyrosine kinase enzymes, instead of non-specifically inhibiting rapidly dividing cells. The crystal structures of related compounds, *viz*, 2-amino-4-(4-pyridyl)pyrimidine and the 1:1 adduct with 4-aminobenzoic acid (Lynch & McClenaghan, 2001), 4-(2-pyridyl)-1H,2*H*-pyrido[1,2-*c*]pyrimidine-1,3-dione (Wolska *et al.*, 2003), 3-(4-methylphenyl)-2-(1-pyridyl)-3*H*-benzo[4,5]furo[3,2-*d*]pyrimidin-4-one (Hu *et al.*, 2006), and 5-phenyl-2-(4-pyridyl)pyrimidine (Santoni *et al.*, 2008) have been reported. In view of the importance of the title compound, its crystal structure is reported.

The title compound crystallizes with two molecules (A & B) in the asymmetric unit (Fig. 1 & 2). The molecular structure consists of an amine nitrogen atom bonded to 5-amino-2-methylphenyl and 3-pyridyl-2-pyrimidine groups, respectively. Bond lengths and bond angles are all within expected ranges (Allen *et al.*, 1987). The dihedral angles between the mean planes of the 2-pyrimidine ring and the phenyl and pyridyl rings are 22.3 (1) and 53.2 (9)° in A, and 6.8 (1) and 11.6 (9)° in B, respectively, presenting a much different structural arrangement in each of these molecules. Crystal packing is influenced by the collective action of weak intermolecular N—H···N hydrogen bond interactions (Table 1 and Fig. 3), π-π stacking interactions between nearby pyridyl A rings, [*Cg*1···*Cg*1^iv^ = 3.8395 Å; slippage = 1.520 Å; *Cg*1 is the centroid of C12A–C15A/N5A/C16A ring; symmetry code: (iv) -*x*, -*y*, 2 - *z*] and C—H···π interactions between the pyridyl A and phenyl A rings and between the phenyl A and B rings (Table 1).

A geometry optimized MOPAC PM3 (Parameterized Model 3) computational calculation (Schmidt & Polik, 2007), *in vacuo*, on each molecule separately supports these observations. The dihedral angle between the mean planes of the 2-pyrimidine ring and the phenyl and pyridyl rings change to 36.86, 40.06° in A and 0.00, 9.95° in B, respectively, providing support to these effects and contribute to the packing of these molecules into chains propagating along the [011].

### Experimental

The title compound was obtained as a gift sample from INTERMED LABS PRIVATE LTD., Bangalore, India. X-ray quality crystals were grown from methanol: ethyl acetate (9:1) by slow evaporation of solvent mixture. The melting range was found to be 398–401 K.

### Refinement

All of the H atoms were placed in their calculated positions and then refined using the riding model, with C—N = 0.88 Å and C—H = 0.95–0.98 Å, and with *U*_iso_(H) = 1.19–1.50*U*_eq_(C, N).

### Crystal data

**Table:**

C_16_H_15_N_5_	*Z* = 4
*M**_r_* = 277.33	*F*(000) = 584
Triclinic, *P*1	*D*_x_ = 1.330 Mg m^−^^3^
Hall symbol: -P 1	Cu *K*α radiation, λ = 1.54184 Å
*a* = 9.2242 (3) Å	Cell parameters from 8631 reflections
*b* = 12.5399 (4) Å	θ = 4.4–73.5°
*c* = 12.8594 (4) Å	µ = 0.67 mm^−^^1^
α = 72.719 (3)°	*T* = 200 K
β = 89.724 (3)°	Prism, yellow-orange
γ = 77.712 (3)°	0.55 × 0.48 × 0.37 mm
*V* = 1385.05 (8) Å^3^	

### Data collection

**Table:**

Oxford Diffraction Gemini R diffractometer	4629 reflections with *I* > 2σ(*I*)
Radiation source: fine-focus sealed tube	*R*_int_ = 0.041
graphite	θ_max_ = 73.7°, θ_min_ = 4.4°
Detector resolution: 10.5081 pixels mm^-1^	*h* = −11→10
φ and ω scans	*k* = −15→15
11476 measured reflections	*l* = −15→15
5337 independent reflections	

### Refinement

**Table:**

Refinement on *F*^2^	Primary atom site location: structure-invariant direct methods
Least-squares matrix: full	Secondary atom site location: difference Fourier map
*R*[*F*^2^ > 2σ(*F*^2^)] = 0.066	Hydrogen site location: inferred from neighbouring sites
*wR*(*F*^2^) = 0.198	H-atom parameters constrained
*S* = 1.08	*w* = 1/[σ^2^(*F*_o_^2^) + (0.1456*P*)^2^ + 0.1456*P*] where *P* = (*F*_o_^2^ + 2*F*_c_^2^)/3
5337 reflections	(Δ/σ)_max_ = 0.008
381 parameters	Δρ_max_ = 0.32 e Å^−^^3^
0 restraints	Δρ_min_ = −0.36 e Å^−^^3^

### Special details

**Table:**

Geometry. All e.s.d.'s (except the e.s.d. in the dihedral angle between two l.s. planes) are estimated using the full covariance matrix. The cell e.s.d.'s are taken into account individually in the estimation of e.s.d.'s in distances, angles and torsion angles; correlations between e.s.d.'s in cell parameters are only used when they are defined by crystal symmetry. An approximate (isotropic) treatment of cell e.s.d.'s is used for estimating e.s.d.'s involving l.s. planes.
Refinement. Refinement of *F*^2^ against ALL reflections. The weighted *R*-factor *wR* and goodness of fit *S* are based on *F*^2^, conventional *R*-factors *R* are based on *F*, with *F* set to zero for negative *F*^2^. The threshold expression of *F*^2^ > σ(*F*^2^) is used only for calculating *R*-factors(gt) *etc*. and is not relevant to the choice of reflections for refinement. *R*-factors based on *F*^2^ are statistically about twice as large as those based on *F*, and *R*- factors based on ALL data will be even larger.

### Fractional atomic coordinates and isotropic or equivalent isotropic displacement parameters (Å^2^)

**Table:**

	*x*	*y*	*z*	*U*_iso_*/*U*_eq_	
N1A	0.45135 (18)	1.19152 (15)	0.78047 (14)	0.0532 (4)	
H1AA	0.4813	1.2472	0.7965	0.064*	
H1AB	0.4989	1.1200	0.8104	0.064*	
N2A	0.09164 (16)	1.06549 (11)	0.59640 (10)	0.0367 (3)	
H2AB	0.0731	1.0718	0.5275	0.044*	
N3A	0.00963 (15)	0.89841 (12)	0.63377 (10)	0.0351 (3)	
N4A	0.09680 (14)	0.95804 (11)	0.77803 (10)	0.0315 (3)	
N5A	0.22563 (16)	0.91112 (13)	1.09733 (11)	0.0402 (3)	
C1A	0.14467 (17)	1.15507 (13)	0.61884 (11)	0.0325 (3)	
C2A	0.27421 (17)	1.12989 (14)	0.68428 (12)	0.0354 (3)	
H2AA	0.3267	1.0525	0.7136	0.043*	
C3A	0.32847 (18)	1.21663 (15)	0.70762 (13)	0.0384 (4)	
C4A	0.2529 (2)	1.33008 (14)	0.66004 (13)	0.0402 (4)	
H4AA	0.2900	1.3908	0.6720	0.048*	
C5A	0.1243 (2)	1.35351 (14)	0.59555 (13)	0.0405 (4)	
H5AA	0.0740	1.4312	0.5644	0.049*	
C6A	0.06427 (18)	1.26855 (14)	0.57380 (11)	0.0349 (3)	
C7A	−0.0847 (2)	1.29936 (16)	0.51227 (14)	0.0449 (4)	
H7AA	−0.1031	1.2322	0.4949	0.067*	
H7AB	−0.0852	1.3614	0.4445	0.067*	
H7AC	−0.1627	1.3245	0.5573	0.067*	
C8A	0.06709 (16)	0.97057 (13)	0.67282 (11)	0.0307 (3)	
C9A	−0.02004 (18)	0.80801 (14)	0.70933 (13)	0.0364 (4)	
H9AA	−0.0606	0.7550	0.6856	0.044*	
C10A	0.00484 (18)	0.78733 (14)	0.81973 (12)	0.0352 (3)	
H10A	−0.0175	0.7222	0.8717	0.042*	
C11A	0.06433 (15)	0.86674 (13)	0.85112 (12)	0.0305 (3)	
C12A	0.09418 (16)	0.85691 (13)	0.96677 (11)	0.0309 (3)	
C13A	0.02152 (18)	0.79443 (15)	1.05046 (13)	0.0379 (4)	
H13A	−0.0485	0.7548	1.0347	0.045*	
C14A	0.05232 (19)	0.79062 (15)	1.15671 (13)	0.0410 (4)	
H14A	0.0043	0.7484	1.2152	0.049*	
C15A	0.15462 (19)	0.84969 (15)	1.17594 (13)	0.0396 (4)	
H15A	0.1758	0.8465	1.2492	0.048*	
C16A	0.19464 (18)	0.91374 (14)	0.99509 (12)	0.0352 (3)	
H16A	0.2441	0.9570	0.9383	0.042*	
N1B	0.6390 (2)	0.87330 (16)	0.93581 (14)	0.0577 (5)	
H1BA	0.7029	0.9169	0.9334	0.069*	
H1BB	0.6078	0.8380	0.9988	0.069*	
N2B	0.35671 (16)	0.69036 (13)	0.74824 (11)	0.0410 (3)	
H2BB	0.3241	0.6949	0.6825	0.049*	
N3B	0.23997 (18)	0.54289 (14)	0.79199 (12)	0.0442 (4)	
N4B	0.33110 (14)	0.60433 (12)	0.93401 (11)	0.0351 (3)	
N5B	0.40511 (18)	0.59479 (14)	1.25219 (12)	0.0479 (4)	
C1B	0.44747 (17)	0.76724 (14)	0.74862 (13)	0.0365 (4)	
C2B	0.49205 (18)	0.78610 (14)	0.84372 (13)	0.0386 (4)	
H2BA	0.4574	0.7484	0.9115	0.046*	
C3B	0.58759 (18)	0.86016 (15)	0.84028 (15)	0.0409 (4)	
C4B	0.63588 (18)	0.91542 (15)	0.73977 (15)	0.0436 (4)	
H4BA	0.7005	0.9661	0.7357	0.052*	
C5B	0.58953 (19)	0.89634 (15)	0.64595 (15)	0.0439 (4)	
H5BA	0.6238	0.9347	0.5783	0.053*	
C6B	0.49516 (18)	0.82354 (15)	0.64661 (13)	0.0402 (4)	
C7B	0.4463 (2)	0.80618 (19)	0.54245 (15)	0.0525 (5)	
H7BA	0.5010	0.8444	0.4824	0.079*	
H7BB	0.3395	0.8388	0.5266	0.079*	
H7BC	0.4665	0.7241	0.5506	0.079*	
C8B	0.30922 (17)	0.61004 (14)	0.82990 (13)	0.0351 (3)	
C9B	0.1970 (3)	0.46071 (19)	0.86902 (16)	0.0539 (5)	
H9BA	0.1491	0.4103	0.8469	0.065*	
C10B	0.2180 (3)	0.44439 (18)	0.97932 (16)	0.0528 (5)	
H10B	0.1892	0.3829	1.0323	0.063*	
C11B	0.28304 (18)	0.52179 (14)	1.00929 (14)	0.0373 (4)	
C12B	0.30358 (18)	0.51873 (14)	1.12446 (13)	0.0374 (4)	
C13B	0.2455 (2)	0.44605 (18)	1.20945 (15)	0.0509 (5)	
H13B	0.1905	0.3952	1.1954	0.061*	
C14B	0.2686 (3)	0.44835 (19)	1.31517 (16)	0.0563 (5)	
H14B	0.2303	0.3990	1.3747	0.068*	
C15B	0.3480 (2)	0.52349 (18)	1.33216 (15)	0.0494 (4)	
H15B	0.3631	0.5248	1.4048	0.059*	
C16B	0.3822 (2)	0.59124 (16)	1.15134 (14)	0.0420 (4)	
H16B	0.4222	0.6416	1.0937	0.050*	

### Atomic displacement parameters (Å^2^)

**Table:**

	*U*^11^	*U*^22^	*U*^33^	*U*^12^	*U*^13^	*U*^23^
N1A	0.0455 (8)	0.0539 (9)	0.0633 (10)	−0.0141 (7)	−0.0140 (7)	−0.0198 (8)
N2A	0.0504 (8)	0.0383 (7)	0.0242 (6)	−0.0166 (6)	−0.0026 (5)	−0.0088 (5)
N3A	0.0419 (7)	0.0404 (7)	0.0270 (6)	−0.0152 (6)	−0.0007 (5)	−0.0119 (5)
N4A	0.0344 (6)	0.0360 (7)	0.0262 (6)	−0.0104 (5)	0.0003 (5)	−0.0107 (5)
N5A	0.0437 (7)	0.0474 (8)	0.0311 (7)	−0.0121 (6)	−0.0043 (5)	−0.0129 (6)
C1A	0.0392 (8)	0.0361 (8)	0.0248 (6)	−0.0131 (6)	0.0039 (5)	−0.0100 (5)
C2A	0.0367 (8)	0.0372 (8)	0.0336 (7)	−0.0106 (6)	0.0018 (6)	−0.0109 (6)
C3A	0.0381 (8)	0.0463 (9)	0.0345 (8)	−0.0159 (7)	0.0019 (6)	−0.0132 (6)
C4A	0.0502 (9)	0.0402 (8)	0.0358 (8)	−0.0195 (7)	0.0028 (6)	−0.0130 (6)
C5A	0.0546 (10)	0.0343 (8)	0.0319 (7)	−0.0109 (7)	−0.0007 (6)	−0.0082 (6)
C6A	0.0413 (8)	0.0394 (8)	0.0238 (6)	−0.0101 (6)	0.0001 (6)	−0.0084 (6)
C7A	0.0495 (9)	0.0448 (9)	0.0381 (8)	−0.0037 (7)	−0.0097 (7)	−0.0135 (7)
C8A	0.0321 (7)	0.0344 (7)	0.0271 (7)	−0.0087 (6)	0.0005 (5)	−0.0107 (5)
C9A	0.0409 (8)	0.0419 (8)	0.0332 (8)	−0.0184 (6)	0.0025 (6)	−0.0153 (6)
C10A	0.0389 (8)	0.0396 (8)	0.0299 (7)	−0.0158 (6)	0.0035 (6)	−0.0097 (6)
C11A	0.0285 (7)	0.0364 (7)	0.0274 (7)	−0.0076 (6)	0.0016 (5)	−0.0106 (6)
C12A	0.0304 (7)	0.0350 (7)	0.0271 (7)	−0.0056 (6)	0.0003 (5)	−0.0100 (6)
C13A	0.0391 (8)	0.0466 (9)	0.0306 (8)	−0.0144 (7)	0.0032 (6)	−0.0121 (6)
C14A	0.0451 (9)	0.0497 (9)	0.0278 (8)	−0.0125 (7)	0.0073 (6)	−0.0099 (6)
C15A	0.0460 (9)	0.0457 (9)	0.0262 (7)	−0.0050 (7)	−0.0026 (6)	−0.0132 (6)
C16A	0.0379 (8)	0.0411 (8)	0.0281 (7)	−0.0123 (6)	0.0003 (6)	−0.0103 (6)
N1B	0.0666 (11)	0.0661 (11)	0.0539 (9)	−0.0375 (9)	0.0047 (8)	−0.0222 (8)
N2B	0.0444 (7)	0.0495 (8)	0.0332 (7)	−0.0205 (6)	0.0025 (5)	−0.0117 (6)
N3B	0.0504 (8)	0.0514 (8)	0.0397 (7)	−0.0241 (7)	0.0054 (6)	−0.0182 (6)
N4B	0.0319 (6)	0.0393 (7)	0.0362 (7)	−0.0123 (5)	0.0042 (5)	−0.0118 (5)
N5B	0.0513 (9)	0.0535 (9)	0.0423 (8)	−0.0134 (7)	−0.0011 (6)	−0.0183 (7)
C1B	0.0322 (7)	0.0371 (8)	0.0401 (8)	−0.0099 (6)	0.0039 (6)	−0.0101 (6)
C2B	0.0376 (8)	0.0407 (8)	0.0397 (8)	−0.0139 (7)	0.0062 (6)	−0.0117 (7)
C3B	0.0355 (8)	0.0400 (8)	0.0490 (9)	−0.0108 (6)	0.0017 (7)	−0.0145 (7)
C4B	0.0354 (8)	0.0401 (8)	0.0570 (10)	−0.0152 (7)	0.0061 (7)	−0.0123 (7)
C5B	0.0384 (8)	0.0433 (9)	0.0461 (9)	−0.0110 (7)	0.0081 (7)	−0.0067 (7)
C6B	0.0362 (8)	0.0424 (9)	0.0392 (8)	−0.0084 (7)	0.0032 (6)	−0.0084 (7)
C7B	0.0612 (11)	0.0611 (11)	0.0365 (9)	−0.0258 (9)	0.0064 (8)	−0.0079 (8)
C8B	0.0314 (7)	0.0384 (8)	0.0374 (8)	−0.0103 (6)	0.0033 (6)	−0.0127 (6)
C9B	0.0702 (12)	0.0605 (11)	0.0472 (10)	−0.0402 (10)	0.0110 (9)	−0.0229 (9)
C10B	0.0721 (13)	0.0568 (11)	0.0424 (9)	−0.0398 (10)	0.0124 (8)	−0.0164 (8)
C11B	0.0350 (8)	0.0407 (8)	0.0383 (8)	−0.0121 (6)	0.0061 (6)	−0.0124 (6)
C12B	0.0357 (8)	0.0403 (8)	0.0376 (8)	−0.0093 (6)	0.0047 (6)	−0.0132 (7)
C13B	0.0584 (11)	0.0587 (11)	0.0436 (10)	−0.0277 (9)	0.0129 (8)	−0.0176 (8)
C14B	0.0692 (13)	0.0637 (12)	0.0393 (9)	−0.0244 (10)	0.0153 (8)	−0.0140 (8)
C15B	0.0547 (10)	0.0569 (11)	0.0365 (9)	−0.0077 (8)	0.0032 (7)	−0.0174 (8)
C16B	0.0441 (9)	0.0460 (9)	0.0379 (8)	−0.0145 (7)	0.0023 (7)	−0.0125 (7)

### Geometric parameters (Å, °)

**Table:**

N1A—C3A	1.394 (2)	N1B—C3B	1.386 (2)
N1A—H1AA	0.8800	N1B—H1BA	0.8800
N1A—H1AB	0.8800	N1B—H1BB	0.8800
N2A—C8A	1.3606 (19)	N2B—C8B	1.365 (2)
N2A—C1A	1.4186 (19)	N2B—C1B	1.406 (2)
N2A—H2AB	0.8800	N2B—H2BB	0.8800
N3A—C9A	1.334 (2)	N3B—C9B	1.328 (2)
N3A—C8A	1.3522 (19)	N3B—C8B	1.353 (2)
N4A—C11A	1.3371 (19)	N4B—C8B	1.333 (2)
N4A—C8A	1.3377 (18)	N4B—C11B	1.342 (2)
N5A—C16A	1.336 (2)	N5B—C16B	1.330 (2)
N5A—C15A	1.338 (2)	N5B—C15B	1.336 (3)
C1A—C2A	1.388 (2)	C1B—C2B	1.392 (2)
C1A—C6A	1.406 (2)	C1B—C6B	1.410 (2)
C2A—C3A	1.397 (2)	C2B—C3B	1.402 (2)
C2A—H2AA	0.9500	C2B—H2BA	0.9500
C3A—C4A	1.396 (2)	C3B—C4B	1.394 (2)
C4A—C5A	1.378 (2)	C4B—C5B	1.384 (3)
C4A—H4AA	0.9500	C4B—H4BA	0.9500
C5A—C6A	1.396 (2)	C5B—C6B	1.387 (2)
C5A—H5AA	0.9500	C5B—H5BA	0.9500
C6A—C7A	1.505 (2)	C6B—C7B	1.505 (2)
C7A—H7AA	0.9800	C7B—H7BA	0.9800
C7A—H7AB	0.9800	C7B—H7BB	0.9800
C7A—H7AC	0.9800	C7B—H7BC	0.9800
C9A—C10A	1.376 (2)	C9B—C10B	1.380 (3)
C9A—H9AA	0.9500	C9B—H9BA	0.9500
C10A—C11A	1.389 (2)	C10B—C11B	1.387 (2)
C10A—H10A	0.9500	C10B—H10B	0.9500
C11A—C12A	1.4776 (19)	C11B—C12B	1.482 (2)
C12A—C13A	1.392 (2)	C12B—C13B	1.387 (2)
C12A—C16A	1.392 (2)	C12B—C16B	1.394 (2)
C13A—C14A	1.382 (2)	C13B—C14B	1.387 (3)
C13A—H13A	0.9500	C13B—H13B	0.9500
C14A—C15A	1.382 (2)	C14B—C15B	1.375 (3)
C14A—H14A	0.9500	C14B—H14B	0.9500
C15A—H15A	0.9500	C15B—H15B	0.9500
C16A—H16A	0.9500	C16B—H16B	0.9500
			
C3A—N1A—H1AA	120.0	C3B—N1B—H1BA	120.0
C3A—N1A—H1AB	120.0	C3B—N1B—H1BB	120.0
H1AA—N1A—H1AB	120.0	H1BA—N1B—H1BB	120.0
C8A—N2A—C1A	125.27 (12)	C8B—N2B—C1B	132.56 (13)
C8A—N2A—H2AB	117.4	C8B—N2B—H2BB	113.7
C1A—N2A—H2AB	117.4	C1B—N2B—H2BB	113.7
C9A—N3A—C8A	115.26 (12)	C9B—N3B—C8B	114.62 (14)
C11A—N4A—C8A	116.85 (13)	C8B—N4B—C11B	116.88 (14)
C16A—N5A—C15A	117.00 (14)	C16B—N5B—C15B	116.92 (16)
C2A—C1A—C6A	121.09 (14)	C2B—C1B—N2B	122.80 (14)
C2A—C1A—N2A	119.94 (14)	C2B—C1B—C6B	120.89 (15)
C6A—C1A—N2A	118.97 (14)	N2B—C1B—C6B	116.30 (14)
C1A—C2A—C3A	120.97 (15)	C1B—C2B—C3B	120.59 (15)
C1A—C2A—H2AA	119.5	C1B—C2B—H2BA	119.7
C3A—C2A—H2AA	119.5	C3B—C2B—H2BA	119.7
N1A—C3A—C4A	120.05 (15)	N1B—C3B—C4B	120.74 (16)
N1A—C3A—C2A	121.35 (16)	N1B—C3B—C2B	120.41 (16)
C4A—C3A—C2A	118.55 (14)	C4B—C3B—C2B	118.76 (16)
C5A—C4A—C3A	119.66 (15)	C5B—C4B—C3B	119.87 (15)
C5A—C4A—H4AA	120.2	C5B—C4B—H4BA	120.1
C3A—C4A—H4AA	120.2	C3B—C4B—H4BA	120.1
C4A—C5A—C6A	123.15 (15)	C4B—C5B—C6B	122.78 (16)
C4A—C5A—H5AA	118.4	C4B—C5B—H5BA	118.6
C6A—C5A—H5AA	118.4	C6B—C5B—H5BA	118.6
C5A—C6A—C1A	116.50 (14)	C5B—C6B—C1B	117.11 (16)
C5A—C6A—C7A	120.24 (15)	C5B—C6B—C7B	121.02 (15)
C1A—C6A—C7A	123.11 (14)	C1B—C6B—C7B	121.86 (15)
C6A—C7A—H7AA	109.5	C6B—C7B—H7BA	109.5
C6A—C7A—H7AB	109.5	C6B—C7B—H7BB	109.5
H7AA—C7A—H7AB	109.5	H7BA—C7B—H7BB	109.5
C6A—C7A—H7AC	109.5	C6B—C7B—H7BC	109.5
H7AA—C7A—H7AC	109.5	H7BA—C7B—H7BC	109.5
H7AB—C7A—H7AC	109.5	H7BB—C7B—H7BC	109.5
N4A—C8A—N3A	125.95 (14)	N4B—C8B—N3B	126.65 (15)
N4A—C8A—N2A	118.49 (13)	N4B—C8B—N2B	120.66 (14)
N3A—C8A—N2A	115.53 (12)	N3B—C8B—N2B	112.68 (14)
N3A—C9A—C10A	123.62 (14)	N3B—C9B—C10B	123.72 (16)
N3A—C9A—H9AA	118.2	N3B—C9B—H9BA	118.1
C10A—C9A—H9AA	118.2	C10B—C9B—H9BA	118.1
C9A—C10A—C11A	116.47 (14)	C9B—C10B—C11B	116.98 (16)
C9A—C10A—H10A	121.8	C9B—C10B—H10B	121.5
C11A—C10A—H10A	121.8	C11B—C10B—H10B	121.5
N4A—C11A—C10A	121.83 (13)	N4B—C11B—C10B	121.01 (15)
N4A—C11A—C12A	115.68 (13)	N4B—C11B—C12B	116.35 (14)
C10A—C11A—C12A	122.49 (14)	C10B—C11B—C12B	122.65 (15)
C13A—C12A—C16A	117.58 (14)	C13B—C12B—C16B	117.07 (15)
C13A—C12A—C11A	122.18 (14)	C13B—C12B—C11B	122.48 (15)
C16A—C12A—C11A	120.21 (13)	C16B—C12B—C11B	120.45 (15)
C14A—C13A—C12A	119.29 (15)	C12B—C13B—C14B	119.26 (18)
C14A—C13A—H13A	120.4	C12B—C13B—H13B	120.4
C12A—C13A—H13A	120.4	C14B—C13B—H13B	120.4
C13A—C14A—C15A	118.45 (15)	C15B—C14B—C13B	118.68 (18)
C13A—C14A—H14A	120.8	C15B—C14B—H14B	120.7
C15A—C14A—H14A	120.8	C13B—C14B—H14B	120.7
N5A—C15A—C14A	123.75 (14)	N5B—C15B—C14B	123.59 (16)
N5A—C15A—H15A	118.1	N5B—C15B—H15B	118.2
C14A—C15A—H15A	118.1	C14B—C15B—H15B	118.2
N5A—C16A—C12A	123.93 (14)	N5B—C16B—C12B	124.47 (16)
N5A—C16A—H16A	118.0	N5B—C16B—H16B	117.8
C12A—C16A—H16A	118.0	C12B—C16B—H16B	117.8
			
C8A—N2A—C1A—C2A	53.0 (2)	C8B—N2B—C1B—C2B	−9.9 (3)
C8A—N2A—C1A—C6A	−127.07 (16)	C8B—N2B—C1B—C6B	168.66 (17)
C6A—C1A—C2A—C3A	0.2 (2)	N2B—C1B—C2B—C3B	177.37 (15)
N2A—C1A—C2A—C3A	−179.91 (14)	C6B—C1B—C2B—C3B	−1.1 (3)
C1A—C2A—C3A—N1A	174.58 (15)	C1B—C2B—C3B—N1B	−176.01 (16)
C1A—C2A—C3A—C4A	−2.6 (2)	C1B—C2B—C3B—C4B	0.6 (3)
N1A—C3A—C4A—C5A	−174.45 (16)	N1B—C3B—C4B—C5B	176.50 (17)
C2A—C3A—C4A—C5A	2.8 (2)	C2B—C3B—C4B—C5B	−0.1 (3)
C3A—C4A—C5A—C6A	−0.5 (3)	C3B—C4B—C5B—C6B	0.1 (3)
C4A—C5A—C6A—C1A	−1.9 (2)	C4B—C5B—C6B—C1B	−0.5 (3)
C4A—C5A—C6A—C7A	173.70 (16)	C4B—C5B—C6B—C7B	179.38 (17)
C2A—C1A—C6A—C5A	2.0 (2)	C2B—C1B—C6B—C5B	1.0 (2)
N2A—C1A—C6A—C5A	−177.91 (13)	N2B—C1B—C6B—C5B	−177.55 (14)
C2A—C1A—C6A—C7A	−173.39 (14)	C2B—C1B—C6B—C7B	−178.88 (16)
N2A—C1A—C6A—C7A	6.7 (2)	N2B—C1B—C6B—C7B	2.6 (2)
C11A—N4A—C8A—N3A	−1.2 (2)	C11B—N4B—C8B—N3B	1.8 (2)
C11A—N4A—C8A—N2A	176.72 (13)	C11B—N4B—C8B—N2B	−179.01 (14)
C9A—N3A—C8A—N4A	0.7 (2)	C9B—N3B—C8B—N4B	−3.2 (3)
C9A—N3A—C8A—N2A	−177.29 (13)	C9B—N3B—C8B—N2B	177.55 (17)
C1A—N2A—C8A—N4A	−1.1 (2)	C1B—N2B—C8B—N4B	9.8 (3)
C1A—N2A—C8A—N3A	176.99 (14)	C1B—N2B—C8B—N3B	−170.97 (17)
C8A—N3A—C9A—C10A	0.0 (2)	C8B—N3B—C9B—C10B	0.9 (3)
N3A—C9A—C10A—C11A	−0.1 (2)	N3B—C9B—C10B—C11B	2.4 (3)
C8A—N4A—C11A—C10A	1.0 (2)	C8B—N4B—C11B—C10B	1.9 (2)
C8A—N4A—C11A—C12A	−178.27 (12)	C8B—N4B—C11B—C12B	−178.07 (13)
C9A—C10A—C11A—N4A	−0.5 (2)	C9B—C10B—C11B—N4B	−3.9 (3)
C9A—C10A—C11A—C12A	178.78 (14)	C9B—C10B—C11B—C12B	176.12 (18)
N4A—C11A—C12A—C13A	156.70 (14)	N4B—C11B—C12B—C13B	173.11 (16)
C10A—C11A—C12A—C13A	−22.6 (2)	C10B—C11B—C12B—C13B	−6.9 (3)
N4A—C11A—C12A—C16A	−21.4 (2)	N4B—C11B—C12B—C16B	−6.2 (2)
C10A—C11A—C12A—C16A	159.27 (15)	C10B—C11B—C12B—C16B	173.81 (18)
C16A—C12A—C13A—C14A	−0.5 (2)	C16B—C12B—C13B—C14B	−0.3 (3)
C11A—C12A—C13A—C14A	−178.66 (14)	C11B—C12B—C13B—C14B	−179.66 (17)
C12A—C13A—C14A—C15A	0.2 (3)	C12B—C13B—C14B—C15B	0.4 (3)
C16A—N5A—C15A—C14A	−0.4 (3)	C16B—N5B—C15B—C14B	0.0 (3)
C13A—C14A—C15A—N5A	0.3 (3)	C13B—C14B—C15B—N5B	−0.2 (3)
C15A—N5A—C16A—C12A	0.0 (2)	C15B—N5B—C16B—C12B	0.0 (3)
C13A—C12A—C16A—N5A	0.4 (2)	C13B—C12B—C16B—N5B	0.2 (3)
C11A—C12A—C16A—N5A	178.63 (14)	C11B—C12B—C16B—N5B	179.52 (16)

### Hydrogen-bond geometry (Å, °)

**Table:**

*D*—H···*A*	*D*—H	H···*A*	*D*···*A*	*D*—H···*A*
N1A—H1AA···N5B^i^	0.88	2.36	3.154 (2)	151
N2A—H2AB···N3A^ii^	0.88	2.11	2.9815 (17)	170
N1B—H1BA···N5A^i^	0.88	2.31	3.130 (2)	155
C2A—H2AA···Cg3	0.95	2.87	3.7834 (19)	162
C14A—H14A···Cg2^iii^	0.95	2.64	3.4709 (18)	146

Symmetry codes: (i) −*x*+1, −*y*+2, −*z*+2; (ii) −*x*, −*y*+2, −*z*+1;  (iii) −*x*, −*y*+2, −*z*+2.
